# Green Synthesis of Titanium Dioxide Nanoparticles Using *Maerua oblongifolia* Root Bark Extract: Photocatalytic Degradation and Antibacterial Activities

**DOI:** 10.3390/ma17235835

**Published:** 2024-11-28

**Authors:** Mamo Dikamu Dilika, Gada Muleta Fanta, Tomasz Tański

**Affiliations:** 1 Department of Chemistry, College of Natural and Computational Sciences, Arba Minch University, Arba Minch P.O. Box 21, Ethiopia; mamo.dikamu@amu.edu.et; 2 Department of Materials Science and Engineering, Adama Science and Technology University, Adama P.O. Box 1888, Ethiopia; 3 Center of Advanced Materials Science and Engineering, Adama Science and Technology University, Adama P.O. Box 1888, Ethiopia; 4 Department of Engineering Materials and Biomaterials, Faculty of Mechanical Engineering, Silesian University of Technology, 44-100 Gliwice, Poland

**Keywords:** antibacterial, green synthesis, photocatalysis, methylene blue, methyl orange

## Abstract

The root bark extract of the *Maerua oblongifolia* plant in the green synthesis of titanium dioxide nanoparticles (TiO_2_ NPs) for photocatalytic degradation of toxic pollutants and antibacterial activities was implemented in this study. The root bark extract served as a novel capping and reducing agent for the first time. Characterization of the TiO_2_ NPs was conducted by using visual observation, ultraviolet visible spectrometry (UV-Vis), Fourier transform infrared spectroscopy (FTIR), X-ray diffraction (XRD), and scanning electron microscopy (SEM) techniques, confirming their successful synthesis. The TiO_2_ NPs exhibited maximum absorbance at 323 nm and an average particle size of 19.58 nm; the conjugations and existences of Ti-O and OH vibrational bands were revealed by the FTIR spectrum. The photocatalytic activities of the TiO_2_ NPs were investigated by using solar irradiation as an energy source for aqueous solutions of methyl orange (MO) and methylene blue (MB) dyes. The TiO_2_ NPs showed strong photocatalytic activities by degrading 97.23% MB and 91.8% MO under optimized conditions. Degradation behavior was investigated by isotherms and kinetics models, with the Langmuir isotherms (R^2^: 0.996, 0.979) and Langmuir–Hinshelwood (R^2^: 0.998, 0.997) highest correlation coefficients for MB and MO, respectively. Moreover, the antibacterial efficacy of the green-synthesized TiO_2_ NPs and the results indicated higher antibacterial activities on Gram-negative bacteria (27 ± 0.52).

## 1. Introduction

Environmental pollutants have emerged due to the effects of natural and artificial elements on the earth’s crust. Among those elements, most pollution is related to anthropogenic activities such as population growth, urbanization, and industrialization, which are the main concerns regarding environmental pollution. Fast industrialization, including textile, food processing, paper, dyeing, and dye manufacturing industries, is the primary reason for environmental pollution by discharging high amounts of organic and inorganic effluents into the environment [1].

Ajmal et al.’s 2018 study stated that more than 7 × 10^5^ tons of dyes are produced annually, and of those, 15–20% are discharged into the environment during and after fabric processing [2,3]. Azo dyes (e.g., methyl orange (MO), methylene blue (MB), etc.) are present in those effluents [3]. These dyes are known to be carcinogenic, mutagenic, and toxic to humans at low concentration levels (ppm-ppb) [4,5,6]. Three methods were utilized to degrade such toxic dyes from the environment, which predominantly focused on chemical, physical, and biological treatments. Among the three techniques, chemical methods, including chemical precipitations, are the most popular and widely used chemical methods for decolorizing MB and MO dyes [4]. Coagulation, flocculation, chemical precipitations, catalytic degradation, and adsorptions are the most common chemical treatment techniques. These methods have a number of limitations, including generating harmful byproducts, requiring adsorbent regeneration and disposal, and being limited to selected agents [6]. Therefore, photocatalytic degradation is a promising technique because it does not generate secondary toxic substances in the environment that convert to non-toxic byproducts.

On the other hand, infectious diseases, which cause a huge number of deaths worldwide, are major challenges facing the medical sector. Environmental samples have revealed the presence of numerous pathogenic microorganisms related to the misuse of antibiotics, resulting in mutations and the generation of antimicrobial-resistant microorganisms [2]. The amalgamation of nanotechnology with environmental pollution plays a critical role in the synthesis of NPs, with various applications including the medicine, biotechnology, food industry, agriculture, transportation, textile, and cosmetics industries. Given this, one of the primary methods employed nowadays is the elimination of these types of environmental contaminants from the surroundings by means of photocatalysis using metal and metal oxide nanoparticles [5]. In the process of extracting dyes from the environment using photocatalytic reactions, contaminants may undergo total degradation in addition to decolorization. TiO_2_ nanoparticles, one type of metal oxide nanoparticle, have drawn a lot of interest because of their exceptional electrical and optical properties, high bandgap energy, excellent photocatalytic response, recyclability, stability, biocompatibility, and non-toxicity, which make them viable options for use in environmental remediation and medical applications [6,7].

Numerous conventional techniques have been used to synthesize TiO_2_ NPs; however, these techniques have involved the use of hazardous chemicals, have longer reaction times, and use high temperatures. The green route is the more suitable option for nanoparticle synthesis since it is a non-toxic, eco-friendly, and cost-effective method compared with chemical and physical methods.

Several researchers successfully synthesized TiO_2_ NPs from various plant extracts, as eco-friendly, like orange peel [8], *Cassia auriculata* leaves [9], and *Jatropha curcas* L. latex [10], but the phytochemical content present in the plants varies from plant to plant, which affects the synthesis processes, shape, and size of nanoparticles. *M. oblongifolia* is a traditionally well-known medicinal plant, known by its common name, Sangana, in the Dawuro, Konta, Gamo, Wolayita, and Gofa zones of Ethiopia [11]. The root part of the plant is used as a detergent for washing clothes in the Dawuro zone, which indicates that the plant has high saponin content and good detergent ability. The applications of saponin-rich plant sources in the preparation of nanoparticles involve controlling the size, and shape and preventing agglomeration and aggregation.

**Figure 1:**
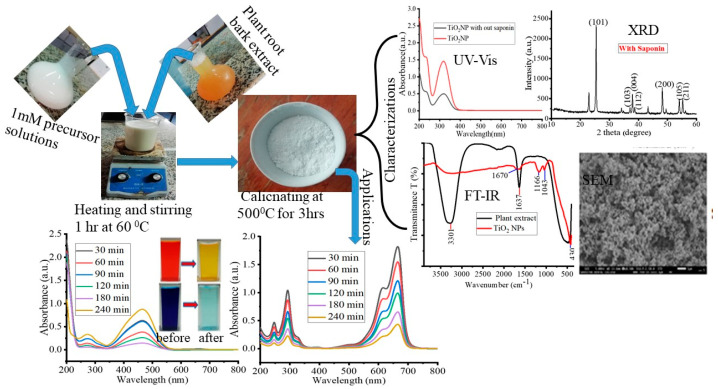
Schematic representation of the work flow of this study.

To the best of our knowledge, this study is the first to employ *M. oblongifolia* root bark extract for photocatalytic degradations and antibacterial activity in the green synthesis of TiO_2_ NPs. The findings of this study might lead to the creation of innovative materials that prevent bacterial infections and dye pollution, improving public health and fostering a cleaner environment. To remove as much of the MB and MO dyes as possible, statistical optimization was performed using the response surface model (RSM) and Box–Behnken design (BBD). Furthermore, the characterization and photocatalytic degradations of both cationic and anionic dyes are expressed in Figure 1.

## 2. Materials and Methods

### 2.1. Materials, Chemicals, and Instruments

The high-purity chemicals and reagents used were of commercial reagent grade, including titanium tetra isopropoxide (97% Ti[OCH(CH_3_)_2_]_4_ MW = 284.22 g/mol), ethanol (97% CH_2_OH, MW = 60 g/mol), sodium hydroxide (97% NaOH, UNI-CHEM), sodium chloride (99.9% NaCl, Riedelde Haen, Seelze, Germany), and n-butanol (98% C_4_H_10_O, Labort fire chemicals, Surat, India). The *M. oblongifolia* root bark used in this study originated from the Dawuro zone, the southwestern region of Ethiopia. A UV-Vis spectrophotometer (SCOROD-50, Mainz, Germany), FTIR (Shimadzu, IR Affinity-1S, Kyoto, Japan), XRD (Shimadzu, XRD-7000, Kyoto, Japan), pH meter (CRISON GLP 22, China), and SEM (JSM-6390, Eindhoven, The Netherlands) were used.

### 2.2. Collection and Preparation of Plant Materials

Root bark of *M. oblongifolia* was collected and immediately transported to the working laboratory of chemistry at Arba Minch University, Ethiopia. To ensure the purity of the sample, the collected sample was subjected to a thorough cleaning process. Subsequently, the cleaned sample was dried under shade for a period of 15 days. Once completely dry, the sample was pulverized using a laboratory grinder, following standard procedures. Refer to Figure S1 for a visual representation of the pulverized sample.

### 2.3. Preparation of Root Bark Extracts

The root bark was extracted by measuring up to 20 g of powdered *M. oblongifolia* root bark and adding 100 mL of distilled water to a 25 mL beaker. After that, the mixes were brought to a boil for 60 min at 55 °C. The deride root extract was boiled and then allowed to cool at room temperature before being filtered. For the next steps, the filtrate was maintained at 4 °C.

### 2.4. Isolations of M. oblongifolia Root Bark Saponin

For the extractions of saponin from the powdered plant materials, 5 g of the materials was combined with 20% ethanol solutions. The mixture was subjected to extractions to obtain a saponin-rich extract, as shown in Figure S2. A rotary evaporator was used to concentrate and dry the recovered components, which were then kept at 4 °C for further study [12].

### 2.5. Green Synthesis of TiO_2_ NPs

The TiO_2_ NP synthesis method was adopted from Sethy et al. [13] with some modifications. One hour of stirring at 60 °C was required after adding 1 mM of titanium tetra isopropoxide to a 250 mL beaker that contained 20 mL of plant root bark extract (with saponin-isolated and without saponin-isolated extracts in a separate beaker). After the reaction was completed, a dark white solution formed, and it was centrifuged for 5 min at 12,000 rpm. Following the centrifugation, ethanol was used to continuously wash and separate the TiO_2_ NPs. Finally, the separated TiO_2_ NPs were left for 12 h at 80 °C in the oven and calcined for 3 h at 500 °C. The procedure is summarized in the Supplementary Materials (Figure S3). Finally, a variety of analytical methods were used to confirm the synthesized nanoparticles, both before and after isolations of the saponin extract.

### 2.6. Characterization of Green-Synthesized TiO_2_ NPs

Various instrumental techniques were used for the characterization of synthesized TiO_2_ NPs. These included a UV-Vis spectrophotometer (SCOROD-50, Mainz, Germany) for spectral and stability studies; FTIR (Shimadzu, IR Affinity-1S, Kyoto, Japan) for functional group studies; XRD (Shimadzu, XRD-7000, Kyoto, Japan) for the crystalline profiles of green-synthesized TiO_2_NPs with the range of 10–70° and resolutions of 20, with CuKα radiations (wavelength of λ = 1.5406 Å) and 40 kV and 40 mA generator voltage and current, respectively; and SEM (JSM-6390, Eindhoven, The Netherlands) for surface morphology and particle size distributions.

### 2.7. The Photocatalytic Degradation of Synthesized TiO_2_NPs

To evaluate the green-synthesized TiO_2_ NP’s photocatalytic potential, MB and MO dye degradation was performed. For the individual batch experiment, two distinct 100 mL beakers containing the MB and MO dye solutions were created. Before being exposed to sunlight on a sunny day for photocatalytic degradation, the solutions were constantly agitated for 30 min in the dark to establish the absorption–desorption stage. During the experiment, the mixture was thoroughly stirred, and the progress of the decolorization process was monitored at a 15 min interval by recording UV-Vis spectra. To serve as a control, a reaction without TiO_2_ NPs was also conducted under the same conditions for both dyes, following the methods described by [14,15]. The obtained data from the degradation process were utilized to calculate degradation efficiency, denoted as “D”. The employed equation followed that by [15].

(1) $$D=\frac{C_{0}-C_{t}}{C_{0}}\times 100$$

where *C*_0_ is the initial concentration of the dye before degradation and *C_t_* is the concentration of the dye after degradation of a specific irradiation time.

#### Optimization Parameters for Photocatalytic Degradations by BBD and RSM

Based on the experimental designs, four independent operating parameters were considered to study the photocatalytic degradation efficiency of TiO_2_ NPs. Design-Expert software v.13.0.5.0 was used to carry out the experimental designs followed by a response surface model (RSM) [16]. In Table 1, the effects of four independent factors (A: irradiation time, B: catalyst dose, C: initial dye concentrations, and D: solution pH) were studied on photocatalysis with percent degradations as a result.

### 2.8. Adsorption Isotherms and Kinetic Study

The adsorption behaviors of the MO and MB dyes were investigated using a range of starting dye concentrations from 20 to 200 ppm, with intervals of 50 ppm. The catalyst dosages for the MB and MO dyes were determined to be 0.035 g and 0.04 g, respectively, based on the optimization research. The adsorption behaviors of the dyes were investigated using three different isotherm models: the Freundlich, Timken, and Langmuir models. These models were used to evaluate how effectively the adsorption processes were described, how well they fit the experimental data, and how stable the models were in characterizing the adsorption processes. The effects of adsorption on the rate at which dye was removed from the solutions were ascertained by conducting tests under optimal circumstances for each parameter. The experiments were carried out continuously at intervals of 15 min. The two kinetic models that were used to correlate and analyze the experimental data were pseudo-first- and pseudo-second-order models. The degradation rate of the MB and MO dyes during the photocatalytic process was shown by these models. The results of the adsorption isotherms and kinetic models helped in gaining an in-depth understanding of the processes and degradation behaviors, whether direct or indirect, of the MB and MO dyes in the presence of the synthesized catalyst.

### 2.9. Recovery of Green-Synthesized Photocatalyst

The model dyes MB and MO were utilized in order to evaluate reusability. After every catalytic test, the activity of the recycled catalyst for a subsequent catalytic process was assessed to determine if the catalyst could be reused [17,18].

### 2.10. Antibacterial Activities

#### 2.10.1. Growth Media and Testing Microorganisms

Muller Hinton Agar plate (Himedia Lab. Mumbai, India) was used to test for antibiotic susceptibility as a medium of microbiological growth. Bacterial strains *Escherichia coli* and *Staphylococcus aureus* were collected from the microbiology laboratory and microbial stock cultures.

#### 2.10.2. Antibacterial Activity

Using an agar well diffusion test, the antibacterial activity of the synthesized TiO_2_ NPs against *S. aureus* and *E. coli* was assessed. The media were sterilized petri plates with Muller–Hinton agar inside of them. On a different plate, *E. coli* and *S. aureus* were consistently swabbed. Holes were created in each plate using a sterilized stainless steel cork borer. TiO_2_ NPs at various concentrations (25, 50, 75, and 100 ppm) were introduced into the holes using a micropipette. This was followed by incubation overnight at 370 °C. The diameters of the inhibition zone surrounding each well were measured with a ruler placed against the inverted Petri dish plate.

## 3. Results and Discussion

### 3.1. Characterization of TiO_2_ NPs

**Figure 2:**
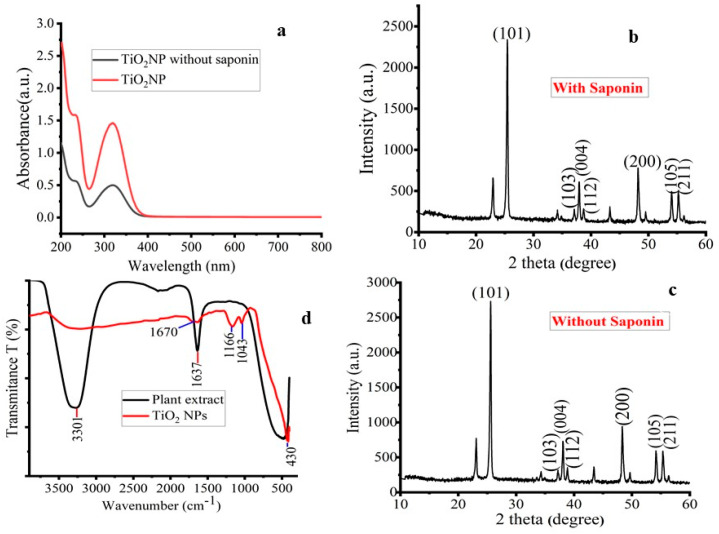
(**a**). Synthesized TiO_2_ NPs; UV-Vis spectrum (**b**). XRD pattern of the TiO_2_ NPs synthesized using saponin-containing extract (**c**). XRD pattern of the TiO_2_ NPs synthesized without saponin extract. (**d**) FTIR spectra of both the *M. oblongifolia* root bark extract and green-synthesized TiO_2_ NPs.

Initially, visual observations were used to record the color changes in the reaction mixtures of titanium tetra isopropoxide solution and aqueous extracts of *M. oblongifolia* root bark. The yellowish root bark extract, when mixed with the watery-white titanium tetra isopropoxide solution, changed color to darkish white, which was the primary indication of the formation of the green-synthesized TiO_2_ NPs. For the green production of TiO_2_ NPs from herbal plant extracts of cassia fistula, a remarkably comparable result was also reported [19]. The color shifts that occur during the synthesis process are shown in Figure S4. The creation of TiO_2_ NPs with the extract containing saponin is depicted in Figure 2a, which shows a strong and intense peak indication at *λ_max_* = 323 nm in the SPR of the synthesized NPs. When the saponin-containing extract was employed, the NPs peak became more intense and pointed than when the root bark extract was used without the saponin-containing extract. This peak clearly shows how saponin regulates the shape and production of NPs. The green-synthesized TiO_2_ NPs’ absorbance spectra were discovered to be similar to those previously published in [20] and at 320 nm [21] and 332 nm [22].

Figure 2b,c above indicate the XRD results of synthesized TiO_2_ NPs before and after isolations of saponin extracted from *M. oblongifolia* root bark extracts. The peaks observed at 25.36°, 37.05°, 37.78°, 38.54°, 48.12°, 54.02°, and 55.04°, which correspond to the miller index planes of 101, 103, 004, 112, 200, 105, and 211, respectively, for both types of nanoparticles, indicate the presences of the anatase phase of TiO_2_ NPs (JCPDS: 84-1286). Similar results were reported with the use of *Azadirachta indicia* leaf extract [23]. The average crystalline particle sizes of the synthesized TiO_2_ NPs were determined to be 24.61 nm and 19.58 nm for the sample before and after isolations of saponin, respectively. This suggests the nanocrystalline nature of the synthesized NPs.

According to the study by Hassan et al. [24], there exists an inverse relationship between the peak intensity and surface functionalization of nanoparticles, implying that the presence of saponin in *M. oblongifolia* root bark extract may have coated the surface of the TiO_2_ NPs, resulting in decreased XRD peak intensity. The findings studied by Hassan et al. [24] indicate that there is an inverse relationship between nanoparticle surface functionalization and peak intensity. This suggests that the saponin in the root bark extract of *M. oblongifolia* may have coated the surface of the TiO_2_ NPs, causing the XRD peak intensity to decrease. The results of the current study on particle size are compared to the previously published literature in Table 2 regarding particle size.

The FTIR spectra of *M. oblongifolia* root bark extracts and the green-synthesized TiO_2_ NPs are shown in Figure 2d. Peaks at 3301 cm^−1^ and 1637 cm^−1^ may be seen in the FTIR spectra of *M. oblongifolia* root bark. The sharp and broadband peak at 3301 cm^−1^ is caused by O-H stretching vibrations of alcohols and phenolic functional groups found in plant extracts [28,29]. The N-H bending vibrations have a peak at 1637 cm^−1^ that represents the amine groups. Peaks at 1670 cm^−1^, 1166 cm^−1^, 1043 cm^−1^, and 430 cm^−1^ may be seen in the FTIR spectra of the green-synthesized TiO_2_ NPs. The inductive double bond stretching in the C=C or C=O ring system is responsible for the FTIR spectrum peak at 1670 cm^−1^. The amine group (C-N) on the TiO_2_ NPs is responsible for the peak at 1166 cm^−1^, while the alkane group on the NPs surfaces is responsible for the peaks at 1043 cm^−1^. Formations of Ti-O and Ti-O-Ti bending were confirmed by the band at 430 cm^−1^ [30].

The presence of metal oxide bonds, Ti-O-Ti, attested to the presence of TiO_2_ in the green-synthesized TiO_2_ NPs. The presence of the Ti-O-Ti bond is due to the strong interaction (capped) of biomolecules with TiO_2_ NPs, which could be due to the presence of saponin, alkaloids, flavonoids, phenols, tannins, steroids, etc., in the plant extracts [20]. As mentioned earlier, the phytochemicals in green synthesis have been deemed responsible for changing the titanium tetra isopropoxide to stable TiO_2_ NPs [31]. The disappearance of the absorbance bands at 3301 cm^−1^ (O-H stretching) in the synthesized TiO_2_ NPs revealed that the phytochemicals play a significant role in controlling and stabilizing metal ions in the synthesis [32]. The spectra of the *M. oblongifolia* root bark extract and synthesized TiO_2_ NPs displayed small shifts, new peaks, and intensity variations.

### 3.2. Mechanism of Green Synthesis of TiO_2_ NPs by Using Plant Extracts

**Figure 3:**
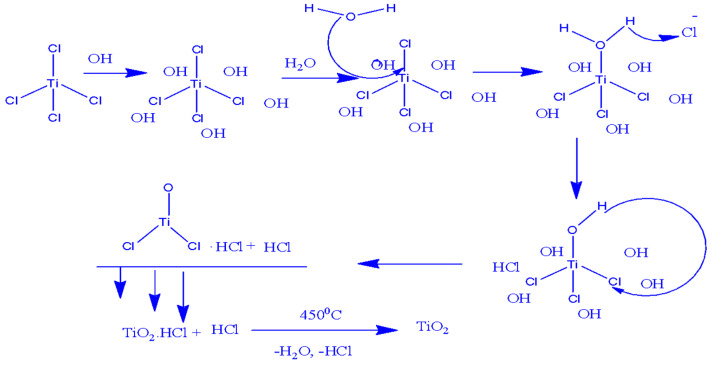
Possible reaction mechanism for the formation of TiO_2_ NPs in the presence of the hydroxyl group (-OH) of the leaf extract of *Jatropha curcas* L. as a capping agent [33].

**Figure 4:**
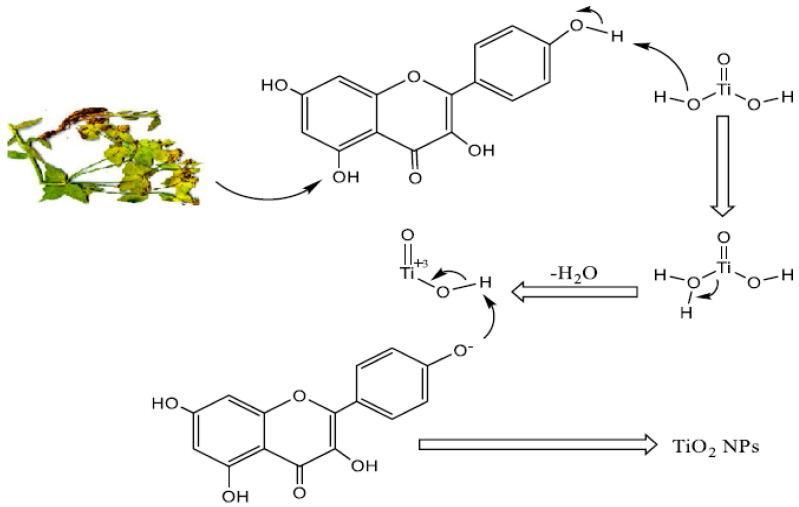
Mechanism of the bioreduction of titanyl hydroxide to TiO_2_ NPs using *Euphorbia hetarade Jaub* root extract [34].

In the abiogenic synthesis of metal oxide nanoparticles, certain phytochemicals found in plant extracts—such as alkaloids, terpenoids, polyphenols, tannins, phenolic acids, and polysaccharides—serve as both bio-reductants and capping agents [33]. Factors including the type and concentration of the plant extract, the concentration of the metal salt, pH levels, temperature, and duration of contact all significantly influence the rate of nanoparticle production, as well as their quantity and characteristics like size and shape. The potential mechanisms by which plant extract interacts with titanium precursors are illustrated in Figure 3 and Figure 4 [33,34].

Regarding the interactions of phytochemicals with metal surfaces, at the nanoscale, an oxide’s band energy is one of its most important components [35]. The energy difference between the full valence band and the empty conduction band is known as the bandgap energy [36]. Additionally, the bandgap of TiO_2_ NPs was determined using a Tauc plot and Equation (2).

(2) $$\left(\alpha hv\right)={A(hv-E_{g})}^{n}$$

where $E_{g}$ = the energy gap, α = the absorption coefficient, h = Planck’s constant, v = the frequency of light, A = the constant of proportionality, and *n* = 1/2 for direct and *n* = 2 for indirect bandgap energy. A Tauc plot $\left(\alpha hv\right)$ is a function of photon energy concerning photon energy hv.

**Figure 5:**
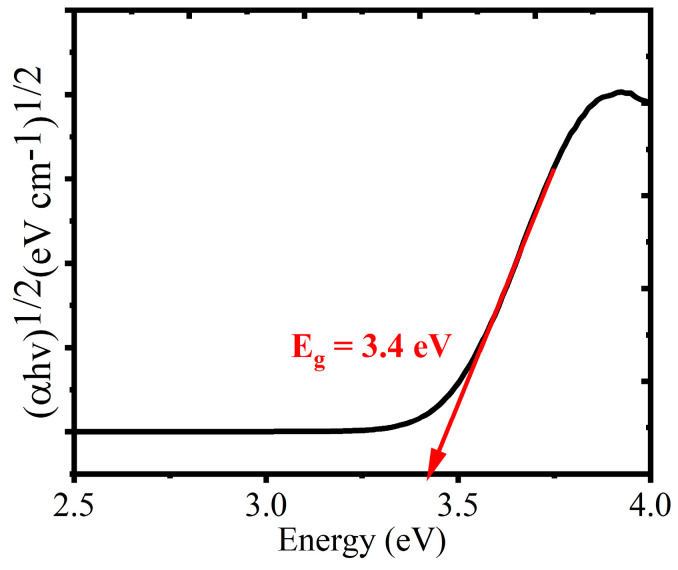
Green-synthesized TiO_2_ NPs’ bandgap energy.

Based on the extrapolation to the linear curve line, the synthesized TiO_2_ NPs’ obtained band energy gap is determined to be 3.4 eV in Figure 5. This agrees with prior research on the *Monsoniaburkeana* plant, which found the energy bandgap for saponin-mediated synthesized TiO_2_ NPs to be 3.53 eV [35].

### 3.3. Surface Morphology

**Figure 6:**
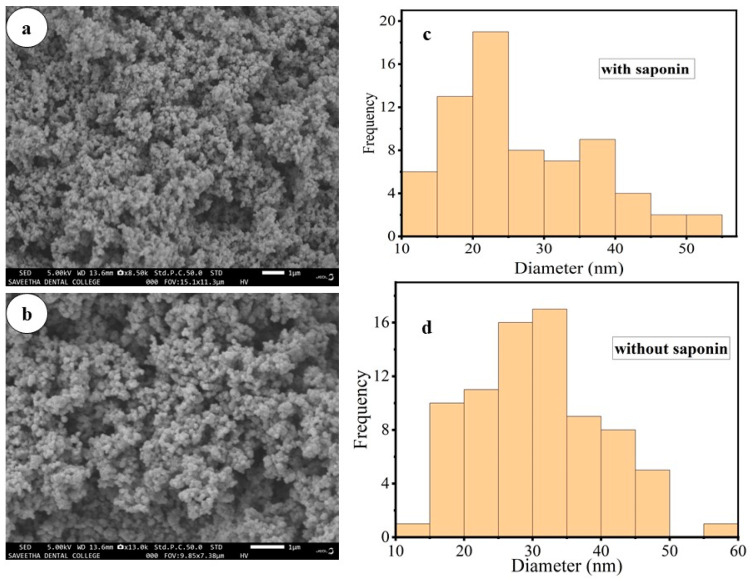
SEM results of the TiO_2_ NPs. (**a**,**b**) indicate before and after isolations of saponins and (**c**,**d**) indicate the particulate size distributions with and without saponin extract, respectively.

SEM images were recorded in order to study the morphology and shape of the synthesized TiO_2_ NPs. From the SEM results, the surface morphology of the green-synthesized TiO_2_ NPs is spherical in shape for both samples, as shown in Figure 6. Additionally, NPs are observed to be agglomerated, which may be the result of interactions and Vander Waals forces among the TiO_2_ NPs [37]. This study revealed that the TiO_2_ NPs synthesized without saponin are more agglomerated and aggregated than those synthesized with saponin. It is evident from this that the saponin found in the plant extract has the ability to stop and control the unwanted growth and enlargement of the particles.

### 3.4. The Mechanism of the TiO_2_ NP Photocatalysis Process

**Figure 7:**
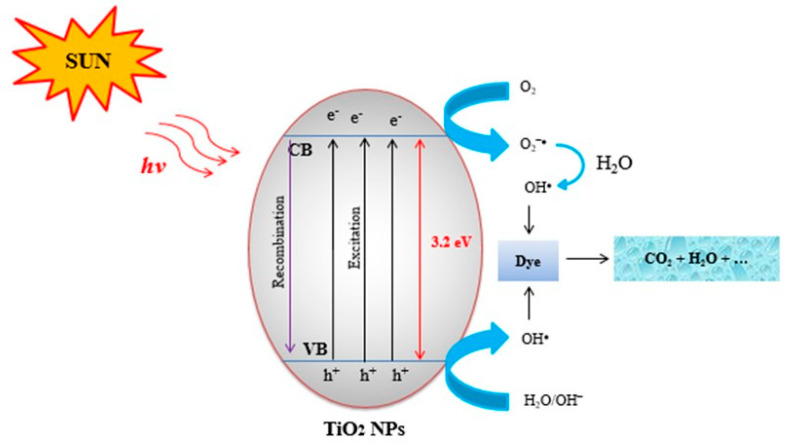
Photocatalytic degradation mechanism over TiO_2_ NPs under sunlight irradiation.

The reaction mechanism of TiO_2_ NPs in photocatalytic degradations has been described by numerous researchers. According to Figure 7, a series of reactions occurred on the surfaces of TiO_2_ NPs through the photocatalytic mechanism. The photons that have energy levels that much or super pass the bandgap energy (3.2 eV), holes (*h*^+^), and elections (e^−^) are created in the valance band (VB) and conduction band (CB), respectively. These holes diffuse to the surfaces of TiO_2_ NPs and participate in the redox reactions of the adsorbed substrates [2,4]. When diffused holes interact with hydroxide ions (OH^−^) or adsorbed H_2_O on the catalyst’s surface, they generate hydroxyl radicals (OH•). Subsequently, dye molecules will react with these produced OH• radicals. This reaction occurs on the surface of the photocatalyst and continues until the organic compound is completely oxidized. The photocatalytic reactions described can be summarized as follows [38]. TiO_2_ + *hν* → e^−^ + *h^+^*e^−^ + O_2_ → O_2_^−^*h^+^* + Organic dyes → CO_2_; *h^+^* + H_2_O → OH• + H^+^ OH + Organic dyes → CO_2_ + H_2_O. This study explored the potential use of green-synthesized TiO_2_ nanoparticles for the degradation of MB and MO dyes.

### 3.5. Evaluations of Photocatalytic Degradation Efficiency

**Figure 8:**
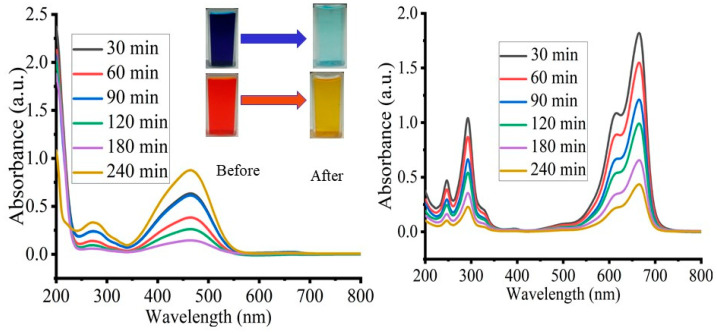
Kinetic study of both MB and MO at optimum conditions.

MB and MO degradation of the dyes under different irradiation sources were used to investigate the photocatalytic activities of synthesized TiO_2_ NPs. The absorption bands of MB and MO were used in this study at 664 and 465 nm, respectively, according to the literature values. Figure 8 illustrates the UV-Vis spectra scanning of MB and MO degradation upon the addition of TiO_2_ NPs under solar irradiation. In both cases listed below, with gradually increasing the irradiation time, the absorption of the dyes at the relevant wavelength gradually decreases.

#### 3.5.1. Effects of Sunlight Irradiation UV-Light (254 Nm) and Catalysts on Degradation

The photocatalyst degradation efficiency was low in this study when there were no light sources because photoelectron and photo hole generations or recombination were insufficient. In a dark condition (catalyst + dye, the previous literature also confirmed that the observed degradation for the dyes in the dark was almost zero [39]. The result supports the previous studies that photodegradation occurs more efficiently in sunlight. The previous literature [40] has shown comparable results for the photocatalytic removal of violet GL_2_B azo dye employing CaO and TiO_2_ NPs. The percentage degradation was calculated in Equation (1) above and is shown in detail in Table S2.

#### 3.5.2. Optimization of the Degradation Process by the Model Equation

In Table 3, we examined the combined effect of four factors on the degradation efficiency of MB and MO: (A) irradiation (min), (B) catalyst dose (g), (C) dye concentration (ppm), and (D) solution pH. A Box–Behnken design (BBD) was used for the four different variables using the surface response model (RSM).

After the experimental data were subjected to regression analysis, the second-order polynomial Equations (3a) and (3b) that were employed in the response surface method provided the expected responses for both MB and MO.

Degradation Efficiency MB (%) = 95.37 + 6.45A + 5.4B−2.04C + 2.2D + 0.627AB − 5.4AC − 3.6 AD +5.6 BC − 5.6BD + 11.48CD − 7.6A2 − 7.6B2 − 3.75C2 − 10.04D2(3a)

Degradation Efficiency MO (%) *=* 75.61 + 0.2475A + 1.71B − 2.6C − 5.10D − 0.01AB + 0.4AC − 2.15AD − 0.4BC − 4.4BD + 0.4CD − 0.2A^2^ − 0.49B^2^ + 1.28C^2^ + 5.6D^2^ (3b)

where A, irradiation time; B, catalyst dose; C, dye concentration; and D, pH of the solution.

#### 3.5.3. ANOVA and Model Adequacy

The lack of fit F-value, which was (4.00, 2.00) for MB and MO, respectively, revealed that the lack of fit was not substantial compared to the simple error. This shows that the chosen model matches the data effectively. For more detailed information, please refer to Table S3a,b. For MB and MO, respectively, the adjusted R^2^ of 0.9898 and 0.9943 and the predicted R^2^ of 0.9726 and 0.9857 in Table 4 were relatively similar, with a difference of less than 0.2 for both dyes. All things considered, this model offers valuable insights into the way factors interact and the deterioration of MB and MO’s parenthood. To obtain further comprehensive details, kindly consult Table S4a,b.

#### 3.5.4. Effects of Different Parameters on Photocatalytic Degradation Efficiency

- Effects of catalyst dosage and irradiation time

**Figure 9:**
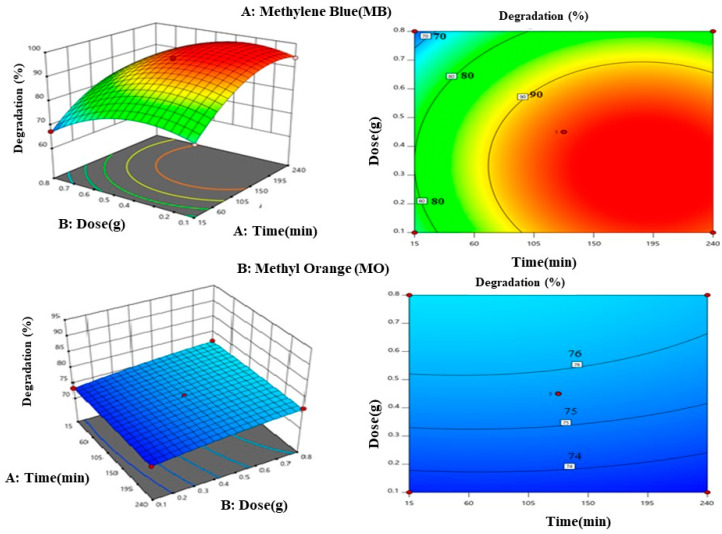
Three-dimensional plot and counterplot for the effects of irradiation time and catalyst dose.

Figure 9 illustrates the interaction effects of catalyst dose and irradiation period on MO and MB degradation efficiency using TiO_2_ NPs. The surface response analysis shows that within the specified ranges of irradiation time, 15–240 min, and catalyst dosage (0.1–0.8 g), the degradation efficiency exhibits a slight increase with both factors. The 3D plot and counterplot demonstrate that the maximum degradation efficiency of 90% is achieved with a catalyst dosage range of 0.36 to 0.6 g and an irradiation time greater than 100 min for MB, while for MO, it reaches 76% with a catalyst dosage range of 0.4–0.6 g and an irradiation time above 150 min. due to the increased number of active sites on the catalyst surface. Prior research has shown that increasing the catalytic dose improves the degradation efficiency of both MB and MO, which causes a rise in the absorption of photons and generations of large quantities of •OH radicals. As a result, this promotes the MB and MO dye molecule degradations [41,42].

b. Effects of pH and Catalytic Dos

**Figure 10:**
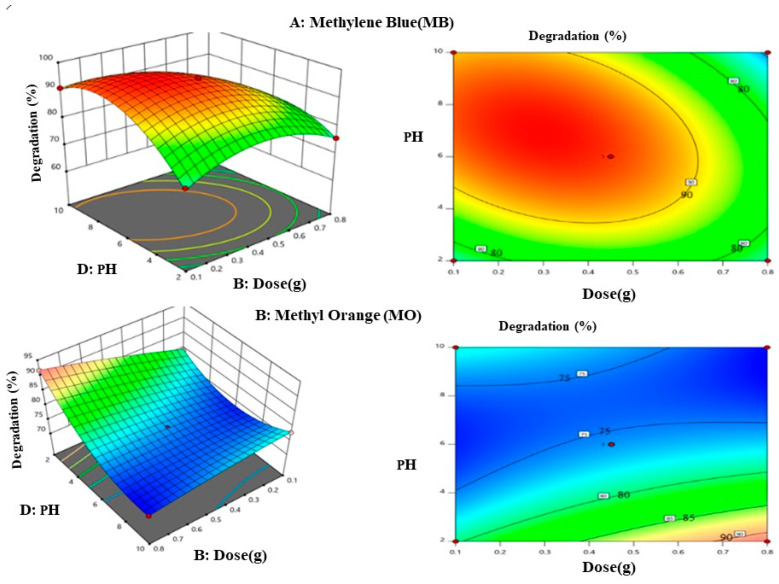
Three-dimensional plot and counterplot for the influence of catalytic dose and pH of the solutions.

The effects of solution pH and catalyst dosage, while maintaining a constant concentration and irradiation period, on the degradation efficiency of MB and MO dyes are shown in Figure 10. At greater catalyst concentrations, the degradation efficiency dropped even more. Interestingly, MO was shown to have a significantly higher degrading efficiency at lower pH levels than MB. These findings imply that pH is an important factor in photocatalytic degradations, with a low pH promoting the breakdown of anionic dyes and a high pH favoring the degradation of cationic dyes. According to [43], these events can be explained by the catalyst surface’s zeta potentials. At an acidic pH, the catalytic surfaces become positively charged, which draws OH ions created when H_2_O_2_ dissociates and speeds up degradation processes. Conversely, at a basic pH, the surface charge on the catalyst becomes negative, leading to electrostatic repulsion and a potential reduction in the efficiency of the degradation processes of MO. The opposite trend is observed for MB.

c. Effect of catalytic dose and initial dye concentration

**Figure 11:**
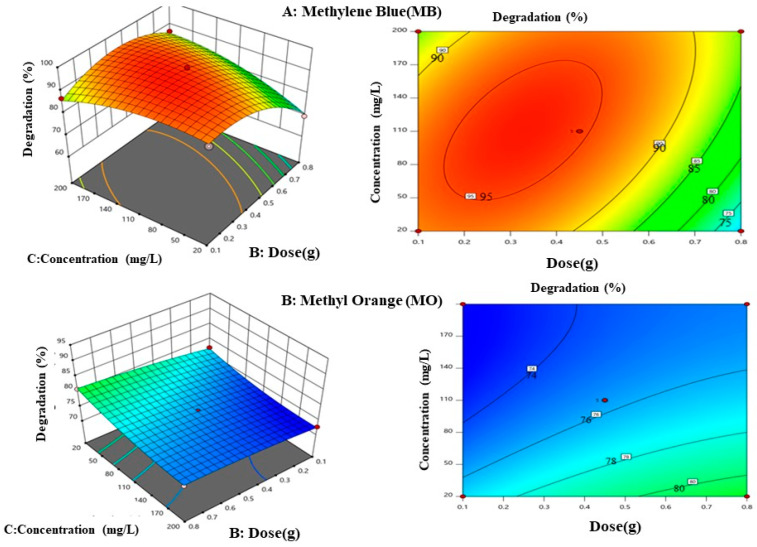
Three-dimensional plot and counterplot for effects of catalytic dose and initial dye concentration.

The catalytic dosage and starting dye concentration interaction effects on the degradations of both MB and MO are depicted in Figure 11, using counterplots and a 3D response surface. Both plots demonstrate that these parameters independently affect the degradation efficiencies of the photocatalyst. At a low initial dye concentration, increasing the catalytic dose increases the degradation; however, at higher initial dye concentrations, efficiency is less noticeable. These behaviors can be explained by the active site accessibility to dye molecules and OH radicals on the surface. Low degradations were seen at low catalytic doses and high initial dye concentrations for both MB and MO, whereas the maximum degradations efficiency was generally attained with a high dose of the photocatalyst and low initial dye concentrations. This aligns with the findings of Oladipo et al. [42].

### 3.6. Experimental Validation of the RSM Model

The model equations were used to determine the optimum catalytic dose, initial dye concentrations, irradiation time, and pH of the solutions that result in maximum dye degradation in 240 min. According to the model predictions in Table 4 above, a maximum dye degradation of 97.23% is achievable with a catalytic does of 0.3681 g at pH of 7.98 and 180.73 min for MB, and 91.8% is achievable with a catalytic dose of 0.4 g achievable at a pH of 2.29 and 172.672 min for MO.

The dye degradation values obtained through experimentation for both MB and MO were 75% and 94.89%, respectively. This indicates a deviation of less than 5% from the estimated value and successfully confirms the achieved optimum operating point. These results suggest that RSM could be a useful tool for improving photocatalytic processes, particularly those involving TiO_2_ NPs. The model that was developed may be applied to each set of given variables in the range to reflect the degradation efficiency of harmful pollutants or to reduce the amount of photocatalyst [44,45].

### 3.7. Adsorption Isotherm Models

This study showed that the correlation between the experimental data and the Langmuir, Freundlich, and Timken isotherm models confirmed the effectiveness of the adsorption process. The Langmuir model suggests that the degradation process might extend beyond adsorbate monolayer coverage across all available homogenous adsorption sites [46,47]. While in the Timken model, which is also governed by the interactions between the adsorbate molecules, in the degradation process, there is a logarithmic decrease with coverage at average concentrations. The Freundlich model is based on empirical equations with the assumptions of heterogeneous surfaces. The Langmuir and Freundlich models were expressed by Equations (4)–(6).

(4) $$R_{L}=\frac{1}{1+C_{0}}$$

(5) $$\log q_{e}=logK_{f}+\frac{1}{nlogC_{e}}$$

where *n* is adsorption intensity and $K_{f}$ is related to the Freundlich constants.

By using linear equations for each model, the completion of monolayer coverage (Langmuir model) and Freundlich equations were validated. The Langmuir equation includes constants related to monolayer capacity and energy of adsorption, while the Freundlich equation involves an empirical parameter related to adsorption intensity. Calculating values 1/*n* from the intercept and slope of $\log q_{e}$ vs. $\log C_{e}$ plots allow for determining the favorability of toxicants’ adsorption.

(6) $$q_{e}=BlnA_{T}+Bln\;C_{e}$$

where *B* = *R_T_/b_T_ A_T_* is Timken equilibrium binding constant L/g, *b_T_* is the Timken isotherm constant, and *B* is the constant related to the heat of sorption (J/mol). From the intercept and the slope, *A_T_* and *b_T_* were determined, respectively, for the plot of *q_t_* against lnt [47]. The Timken equation incorporates equilibrium binding constants and constants related to the heat of sorption, with values obtained from plots of adsorption quantity against the natural logarithm of time. The feasibility of the isotherms was assessed by calculating separation factors, represented as 1/*n*. The calculated separation factor values (*R_L_*) in Equation (4) (0.0209 for MB and 0.00288 for MO) indicated good adsorption for both toxicants. This means that the value of *R_L_* was between 0 and 1 for adsorbents; the parameters corresponding to the Langmuir, Freundlich, and Timken isotherms are listed in Table 5.

According to the study findings, the Langmuir isotherm has a higher correlation regression coefficient for MB (0.996) and MO (0.979) on TiO_2_ NPs than the isotherm models proposed by Freundlich and Timken. The formation of strong monolayer and homogenous surfaces during the dye adsorption processes on the photocatalyst provided in Figure S5 was indicated by the higher regression coefficient. The experimental result indicates that the Langmuir isotherm model matches the equilibrium data better than the Freundlich and Timken isotherm models. The Timken isotherm model identifies the hats of the sorption, which can be used to explain the degradation processes’ *A_T_* and *B_T_* values, were 0.078, 17.35 L/mg and 29.65, 16.72 J/mol for MB and MO, respectively.

### 3.8. Kinetics Study

The kinetics of the dyes’ degradation were investigated to ascertain if the dynamics of the dye degradations of MB and MO ions onto TiO_2_ NPs as adsorbents were accepted. According to the experimental findings, the Langmuir–Hinshelwood (L–H) kinetic model fit the corresponding data better than Equation (7) during the TiO_2_ photocatalytic oxidation of various organic contaminants.

(7) $$\ln(\frac{C_{0}}{C_{t}})=Kt$$

**Figure 12:**
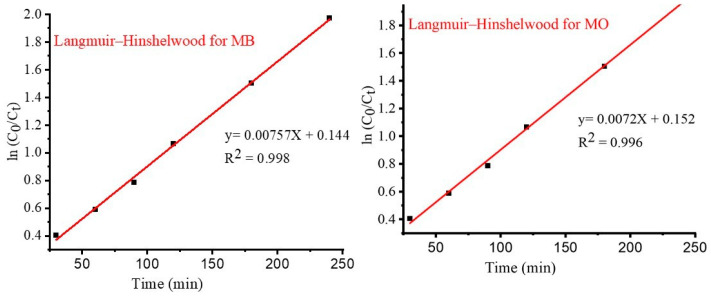
Langmuir–Hinshelwood kinetic degradation models for MB and MO dyes.

**Figure 13:**
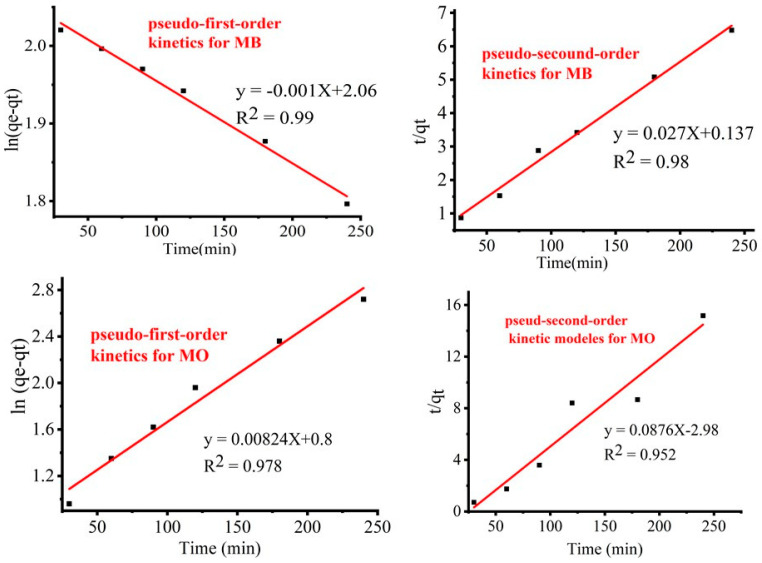
Kinetic models for the degradations of MB and MO.

Equation (7) can describe the relationship between the initial concentration of MB and MO separately (*C*_0_) and the concentration at various times during degradation (*C_t_*). The rate constant (*K*) follows a pseudo-first-order behavior. Figure 12 and Figure 13 show the linear relationship of ln (*C*_0_/*C_t_*) versus irradiation time for both MB and MO.

(8) $$\log\left(q_{e}-q_{t}\right)=log\;q_{e}-\frac{k1}{2.304}\;t$$

When $\log\left(q_{e}-q_{t}\right)$ against *t* is plotted, a linear relationship should result, from which the slope and intercept may be determined. On the other hand, the pseudo-second-order reactions are listed in the lists below.

(9) $$\frac{t}{q_{t}}=\frac{1}{k_{2}{q_{e}}^{2}}-\frac{1}{q_{e}}t$$

where *q_e_* indicates the dye concentration degraded at equilibrium, mg/g, and *k*_2_ represents the rate constant at equilibrium, g/mg min. The pseudo-first- and pseudo-second-order kinetics of the MB and MO dyes, as well as the experimental Langmuir–Hinshelwood kinetic plot, are shown in Figure 12 below. The constants are separately calculated based on the slope and intercept *t/q_t_* against *t* liner graph. Linear graphs of ln (*C*_0_*/C_t_*) versus *t*, the pseudo-first-order model ln (*q_e_ – q_t_*) against *t*, and the straight-line plots of *t/q_t_* against *t* for the pseudo-second-order model are examples of the Langmuir–Hinshelwood method. The higher correlation coefficient, or R^2^ value, was found using the Langmuir–Hinshelwood method and was achieved for both dyes, MB and MO, compared to the pseudo-first- and pseudo-second-order values of 0.998 and 0.997. The outcome suggested that the Langmuir–Hinshelwood method was more precisely followed by the adsorption processes.

The findings are presented in Table S5. Kinetic constants for K, K_1_, and K_2_ were computed and found to be 1.74 × 10^−4^, 4.17 × 10^−6^ min^−1^, and 5.33 × 10^−7^ min^−1^ for the pseudo-first and pseudo-second Langmuir–Hinshelwood kinetic models for MB, respectively, and 3.2 × 10^−4^, 3.43 × 10^−5^, and −2.57 × 10^−3^ for MO. The higher the kinetic rate constant values, the more closely they resemble the experimental values in comparison to the pseudo-first- and pseudo-second-order Langmuir–Hinshelwood values. In Table 6, this finding suggests that Langmuir–Hinshelwood is correlated with the adoration effectiveness of TiO_2_ NPs in both the MB and MO data. This outcome is in good agreement with the dye degradations achieved by photocatalytic dye degradation employing biosynthesized TiO_2_ NPs [35].

### 3.9. Stability Studies

Using UV-Vis spectrum measurements at various time intervals, the stability of the synthesized TiO_2_ NPs was evaluated. No discernible change in the color intensity, spectral peak location, or absorbance during 150 days was observed (Figure S6). The TiO_2_ NPs produced in this study are consistent and reliable and can be safely stored in a dark environment for up to 150 days without deterioration.

### 3.10. Recycling the Photocatalyst

**Figure 14:**
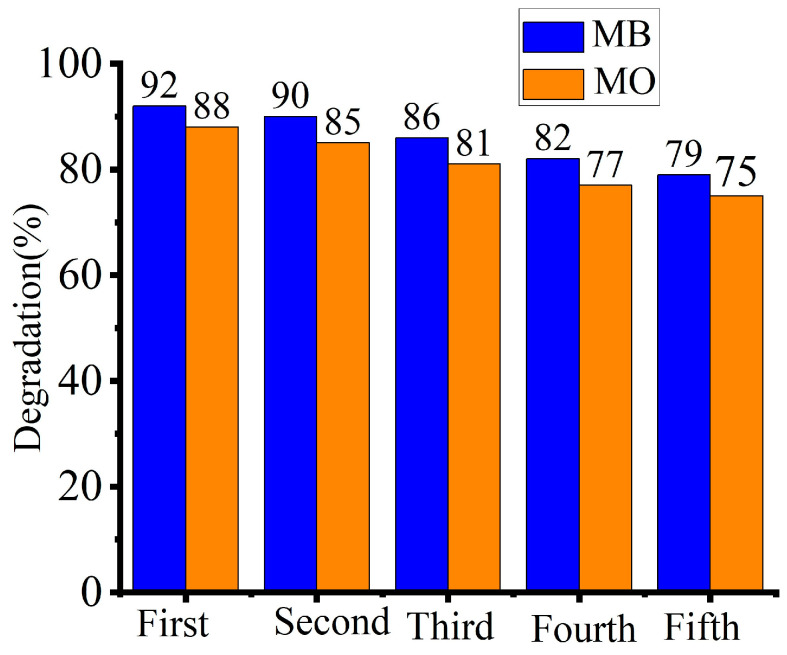
Reusability runs of the catalyst for both MB and MO dyes.

By evaluating the recycled catalyst activities for the ensuring catalytic processes, the photocatalyst recycling capacity was ascertained [17]. The percentages of MB degradation steadily decreased from 92% to 79% based on the recyclability experiment shown in Figure 14. In contrast, the degradation efficiency of MO decreased from 88% to 75% during the first run to the fifth run, respectively; detailed calculations are provided in Table S6 based on Equation (1). Since it is almost difficult to recover all of the photocatalyst after use, some of it may be lost during the recycling process, which accounts for the lower effectiveness of the recycled catalyst. Additionally, the photocatalytic activities of TiO_2_ NPs are reduced when dye molecules block the active pores available on the photocatalytic surfaces [43,52]. The present study found that green-synthesized TiO_2_ NPs from *M. oblongifolia* root bark extract are the best candidates for degrading cationic and anionic dye wastes generated from different sources.

### 3.11. Antibacterial Studies

**Figure 15:**
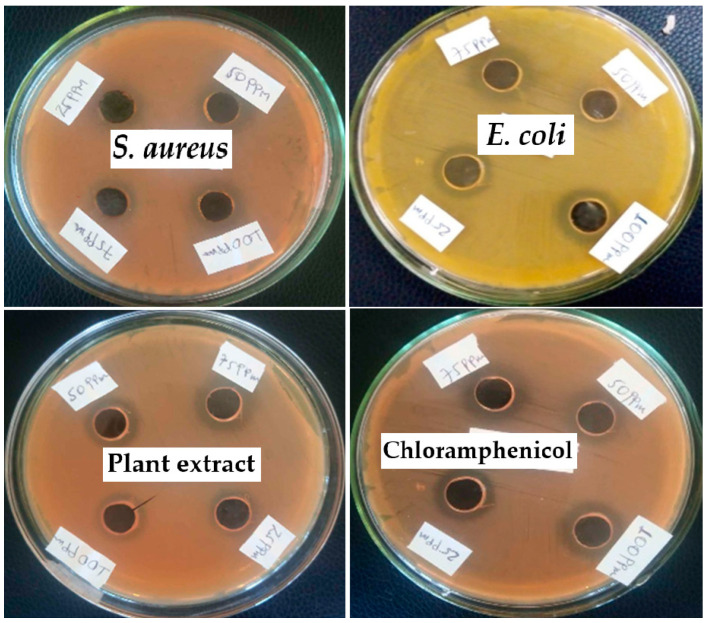
Antibacterial activity (zone of inhibition): images of TiO_2_ nanoparticles, the positive control, and the plant extract against the pathogens *S. aureus* and *E. coli* at different concentrations.

The antibacterial properties of the green-synthesized TiO_2_ NPs were assessed against *S. aureus* and *E. coli* using the agar well diffusion technique. The zone of inhibition around the bacterial growth after 24 h at 37 °C is shown in Table 7 and Figure 15. TiO_2_ NPs have better antibacterial activity against *E. coli* (27 ± 0.61 mm) than *S. aureus* (12 ± 0.34 mm) at 100 ppm. Chloramphenicol (positive control) displayed high antibacterial efficiency against *E. coli* (25 ± 0.4 mm) compared to *S. aureus* (20 ± 0.43 mm) at 100 ppm. This suggests that because Gram-negative bacteria, due to their thinner cell wall, are more susceptible to TiO_2_ photocatalysts, green-synthesized TiO_2_ NPs are particularly effective against them. ROS generation upon light exposure damages bacterial membranes, causing cell death. Similar findings have been reported previously [35]. In addition, the shape and morphology of TiO_2_ NPs influence their antibacterial performance by affecting factors like surface area, light absorptions, ROS generations, and interaction with bacterial cells. Tailoring these characteristics can optimize TiO_2_ NPs for specific antibacterial applications.

Various forms of reactive oxygen species (ROS), such as superoxide (O_2_^−^), peroxide (O_2_^−2^), hydrogen peroxide (H_2_O_2_), and hydroxyl radicals (OH.), play a crucial role in the antibacterial properties of nanoparticles. When nanoparticles come into contact with bacterial cells, they generate ROS, leading to damage to the bacterial cell walls and membranes. The production of ROS is influenced by factors such as the type of nanoparticles used, their size, and their concentration. At very low concentrations, nanoparticles may generate inadequate levels of ROS, resulting in insufficient damage to bacteria [53].

## 4. Conclusions

The TiO_2_NPs were successfully synthesized through a green method by utilizing the root bark extract of *M. oblongifolia* and used for photocatalytic degradations of toxic organic pollutants and antibacterial activities. Visual observations, FTIR, XRD, SEM, and UV-Vis spectroscopy were used to verify the synthesized TiO_2_ NPs for the structural and bandgap energy displayed at 323 nm and 3.4 eV in the Tauc plot. The functional groups of the metal oxide bond on the nanoparticle’s surfaces were demonstrated using FT-IR. Using Scherer equations, the crystalline size was estimated to be 19.58 nm. The SEM revealed that the TiO_2_ NPs were spherical in shape. The ideal conditions for photocatalytic degradations were determined. The TiO_2_ photocatalyst was efficient in the degradations of MB (97.23%) and MO (91.8%). The synthesized TiO_2_ NPs showed remarkable stability and recyclability; even after five cycles, the dye degradation capacity of 79% of the catalytic activity remained constant for the 5 months that were observed. Ultimately, the disc diffusion approach was used to measure the antibacterial efficacy of TiO_2_ NPs at different concentrations, and results revealed a stronger antibacterial activity against *E. coli* than *S. aureus*. Finally, the synthesized TiO_2_ NPs can be utilized as an efficient and affordable photocatalyst for rapid degradations of synthetic dyes in aqueous solution as well as an antimicrobial for microbial-resistant bacteria.

## Figures and Tables

**Table 1:** Independent factors employed for photocatalytic degradations.

Independent Variables	Symbols	Ranges and Levels		
		Low	Middle	High
Irradiation time (minutes)	A	15	127.5	240
Catalyst dose (g)	B	0.1	0.45	0.8
Initial dye concentration (mg/L)	C	20	110	200
pH	D	2	6	10

**Table 2:** Comparisons of green-synthesized TiO_2_ NPs with previous studies.

Plant Used	Temperature	Particle Size (nm)	References
*Jatropha curcas* L. latex	50 °C	25 (XRD)	[10]
*Nyctanthes arbor-tristis leaves*	50 °C	100 (XRD)	[25]
*Eclipta prostrata* leaf	25 °C	49 (XRD)	[26]
*Annona squamosal* peel	60 °C	26 (XRD)	[27]
*cassia auriculata* leaves	-	38 (XRD)	[9]
*M. oblongifolia* root bark before isolating saponin extract	-	19.58 (XRD)	This study
*M. oblongifolia* root bark after isolating saponin extract	-	24.61 (XRD)	This study
*Orange peel*	-	19 (XRD)	[8]

**Table 3:** The experimental data and the obtained results of the MB and MO dyes.

Experimental Conditions					Degradation Efficiency (%)			
					MB		MO	
Run	A	B	C	D	Expt.	Predicted	Expt.	Predicted
1	127.5	0.45	200	10	97	97.31	74.96	75.15
2	127.5	0.1	110	10	91	90.91	78	78.24
3	240	0.1	110	6	94.2	94.59	73.12	73.02
4	15	0.45	200	6	81.77	83.01	74.3	74.23
5	127.5	0.45	110	6	95.85	95.37	75.65	75.3
6	127.5	0.1	20	6	92	92.96	77.2	76.88
7	15	0.45	110	10	76	75.07	78.07	78.67
8	240	0.8	110	6	83	82.5	76.6	76.34
9	15	0.8	110	6	67.33	66.85	77.12	76.69
10	127.5	0.45	110	6	95	95.37	75.9	75.61
11	15	0.1	110	6	76.02	76.43	73.42	73.47
12	15	0.45	110	2	64.2	63.41	83.88	84.16
13	240	0.45	110	2	86.4	87.56	88.2	87.96
14	240	0.45	20	6	97.23	95.82	78	78.67
15	127.5	0.45	200	2	69.12	69.93	84.6	84.52
16	127.5	0.45	20	2	89.2	88.80	91	90.56
17	15	0.45	20	6	67.54	68.06	79.9	79.97
18	127.5	0.45	110	6	95.5	95.37	75.9	75.61
19	127.5	0.8	110	2	75.75	75.67	91.8	91.86
20	127.5	0.45	110	6	94.5	95.37	75.31	75.61
21	127.5	0.8	200	6	86.95	86.22	74.79	75.06
22	127.5	0.8	20	6	69.7	70.90	81	81.18
23	127.5	0.8	110	10	68.3	68.86	73.01	72.92
24	240	0.45	200	6	89.73	89.05	74	74.25
25	127.5	0.45	110	6	96	95.37	75.3	75.61
26	127.5	0.45	20	10	71.16	70.26	79.77	79.57
27	127.5	0.1	200	6	86.8	85.82	72.73	72.51
28	240	0.45	110	10	83.7	84.72	73.8	73.47
29	127.5	0.1	110	2	76.02	75.29	79.3	79.42

Expt. = experimental value.

**Table 4:** Model adequacy.

Dyes	Std.Dev.	Mean	C.V.%	R^2^	Adjusted R^2^	Predicted R^2^	Adeq Precision
MB	1.10	83.34	1.32	0.9949	0.9898	0.9726	42.8642
MO	0.3905	78.16	0.4996	0.9972	0.9943	0.9857	68.9120

**Table 5:** Parameters for the isotherms of MB and MO degradations onto green-synthesized TiO_2_NPs.

Isotherm Model	Parameters	MB	MO
Langmuir	q_max_ (mg/g)	142.85	0.465
	RL	0.0412	0.00029
	R^2^	0.996	0.979
Freundlich	1/*n*	0.99047	1.266
	K_f_ (mg/g)	0.985	1.2835
	R^2^	0.993	0.962
Timken	K_T_ (L/mg)	0.078	17.35
	B_T_ (J/mol)	29.65	16.72
	R^2^	0.981	0.95

**Table 6:** Comparison of photocatalytic degradations of the previous green-synthesized TiO_2_ NPs with the present study.

Plant Species	Light Sources	Dye	Concertation	Time (min)	Dose	Degradation (%)	References
*Jatropha curcas*	Sunlight	CV	7 ppm	300	500 mg	76	[10]
*Carica papaya leaves*	UV	RO	4 ppm	180	25 mg	91	[48]
*A. indica*	Sunlight	MB	10 ppm	360	50 mg	99	[49]
*Sargassum myriocystum*	Sunlight	MB	-	45	-	93	[50]
*Monsonia Burkeana*	UV, 150W	MB	20 ppm	120	60	85%	[35]
*Parthernium hysterophorus*	UV	MO	1 ppm	360	10 mg	82	[51]
*M. oblongifolia*	Sunlight	MB	200 ppm	127.5	0.45 g	97	This study
*M. oblongifolia*	Sunlight	MO	110 ppm	127.5	0.45 g	91	This study

where CR: Crystal Violet, MB. methylene blue, MO: methyl orange, RO: Reactive Orange.

**Table 7:** Zone of inhibition of the synthesized TiO_2_ NPs and chloramphenicol at different concentrations.

Concentration	TiO_2_ NPs *		TiO_2_ NPs **		Chloramphenicol	
	*E. coli*	*S. aureus*	*E. coli*	*S. aureus*	*E. coli*	*S. aureus*
25 ppm	12 ± 0.34	9 ± 0.2	7 ± 0.28	5 ± 0.33	8 ± 0.12	10 ± 0.51
50 ppm	18 ± 0.21	14 ± 0.46	11 ± 0.36	8 ± 0.37	13 ± 0.2	12 ± 0.38
75 ppm	21 ± 0.47	16 ± 0.45	15 ± 0.48	12 ± 0.68	19 ± 0.53	14 ± 0.42
100 ppm	27 ± 0.52	22 ± 0.61	18 ± 0.38	13 ± 0.18	25 ± 0.4	20 ± 0.43

where TiO_2_ NPs * and TiO_2_ NPs ** are nanoparticles synthesized before and after the isolation of saponin extract, respectively.

## Data Availability

The original contributions presented in this study are included in the article/Supplementary Materials. Further inquiries can be directed to the corresponding author.

## Supplementary Materials

The following supporting information can be downloaded at https://www.mdpi.com/article/10.3390/ma17235835/s1: Figure S1: Collections and preparations of plant materials, Figure S2: Extraction and isolations of saponin from the root bark of *M. oblongifolia*, Figure S3: Saponin-mediated syntheses of TiO_2_ NPs from *M. oblongifolia* root bark extract, Figure S4. (a) Plant root bark extract. (b) Titanium (IV) isopropoxide. (c) TiO_2_ NPs, Figure S5: Degradation isotherm models of Freundlich, Langmuir, and Temkin for the degradations of the MB and MO dyes, Figure S6: Stability study of saponin-mediated synthesized TiO_2_NPs within five consecutive months, Table S1. Detailed calculations of the XRD data of TiO_2_ NPs, Table S2: Effects of sunlight irradiation, UV-light (254 nm), and the catalyst on the degradation efficiencies of both MB and MO with the absence and presence of a catalyst, Table S3a,b ANOVA analysis of variance (ANOVA) for the quadratic model of MB and MO, respectively, Table S4a,b, Model adequacies for both MB and MO, respectively, Table S5: Kinetic parameters for the degradations of MB and MO onto green-synthesized TiO_2_ NPs, Table S6: Recycling of the photocatalyst.
