# Supplementary material for: Green Synthesis of Titanium Dioxide Nanoparticles Using Maerua oblongifolia Root Bark Extract: Photocatalytic Degradation and Antibacterial Activities
Source: Materials (Basel). 2024 Nov 28;17(23):5835. doi: 10.3390/ma17235835 (PMC11641925; doi:10.3390/ma17235835)
Supplement: Supplementary file 1 [file materials-17-05835-s001.zip › materials-3328400-supplementary.pdf]

SUPPLEMENTARY MATERIALS

Green Synthesis of Titanium Dioxide Nanoparticles Using *Maerua oblongifolia* Root Bark Extract: Photocatalytic Degradation and Antibacterial Activities

Mamo Dikamu Dilika [1], Gada Muleta Fanta [1,2,3,*] and Tomasz Tański [4,*]

[1] Department of Chemistry, College of Natural and Computational Sciences, Arba Minch University, Arba Minch P.O. Box 21, Ethiopia
[2] Department of Materials Science and Engineering, Adama Science and Technology University, Adama P.O. Box 1888, Ethiopia
[3] Center of Advanced Materials Science and Engineering, Adama Science and Technology University, Adama P.O. Box 1888, Ethiopia
[4] Institute of Engineering Materials and Biomaterials, Faculty of Mechanical Engineering, Silesian University of Technology, 44-100 Gliwice, Poland
* Correspondence: gada.muleta@astu.edu.et (G.M.F.); tomasz.tanski@polsl.pl (T.T.)

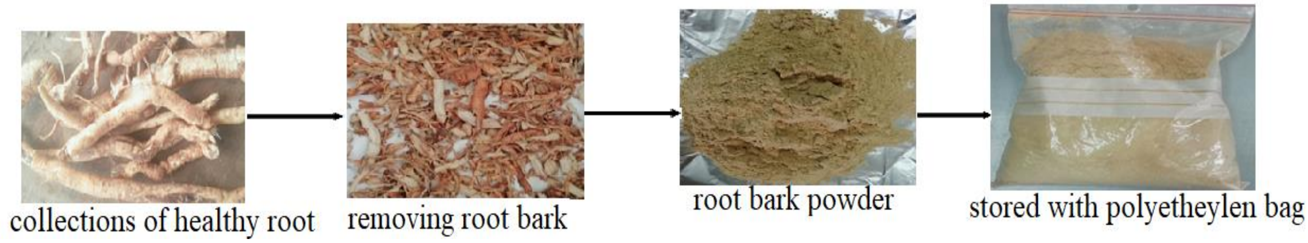

Figure S1. Collections and preparations of plant materials

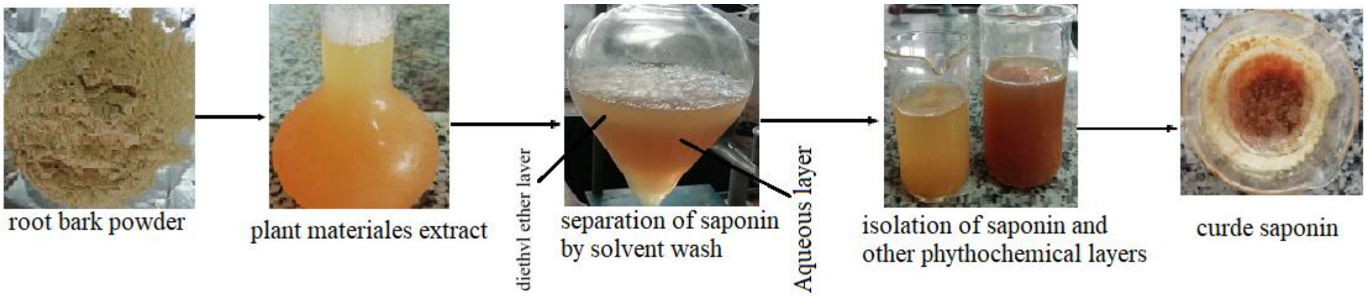

Figure S2. Extraction and isolations of saponin from root bark of M. oblongifolia.

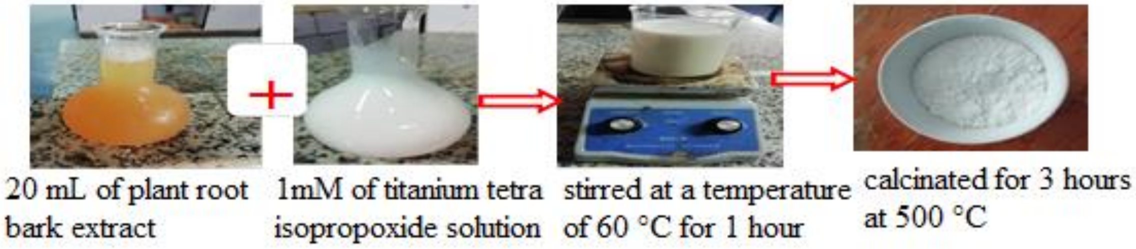

Figure S3. Saponin mediated syntheses of TiO_2 NPs from *M. oblongifolia* root bark extract.

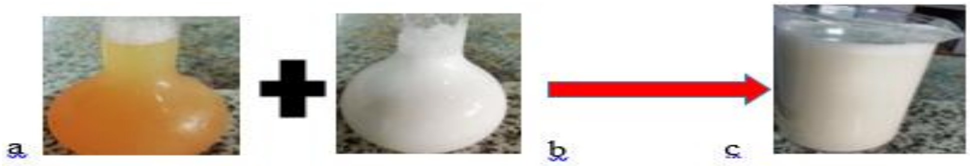

Figure S4. (a) Plant root bark extract. (b) Titanium (IV)isopropoxide. (c) TiO_2 NPs

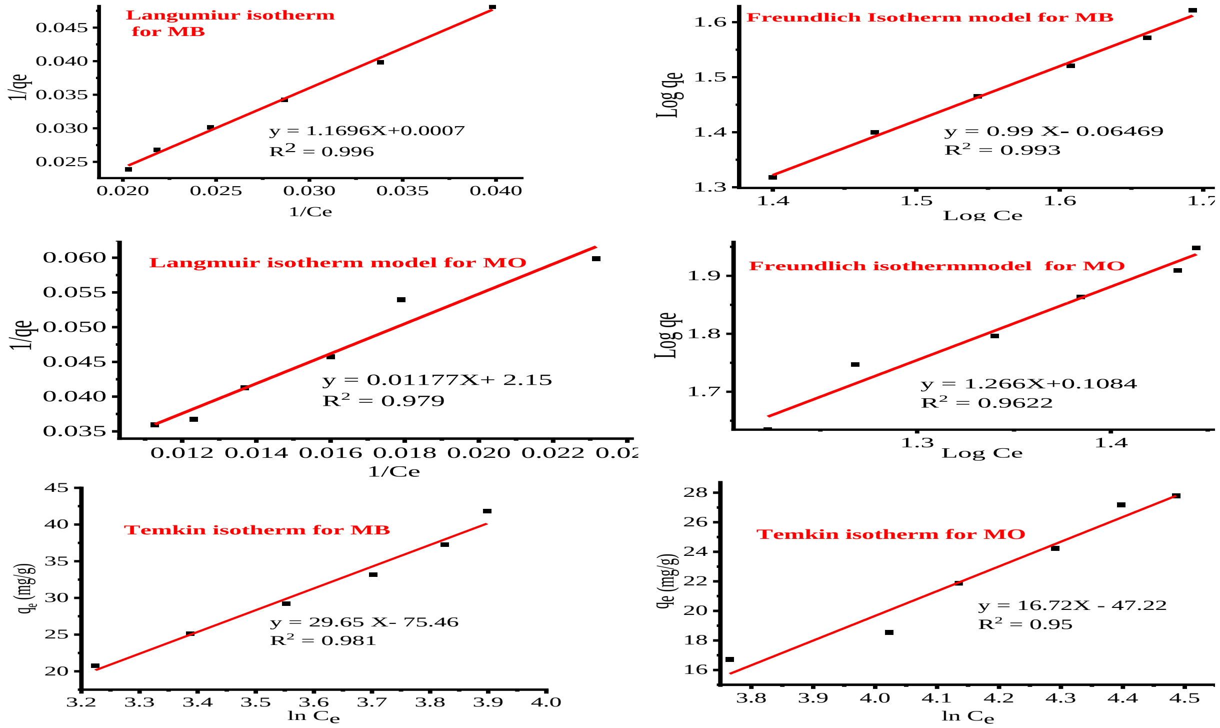

Figure S5. Degradation isotherm models of Freundlich, Langmuir, and Temkin for the degradations of MB and MO dyes.

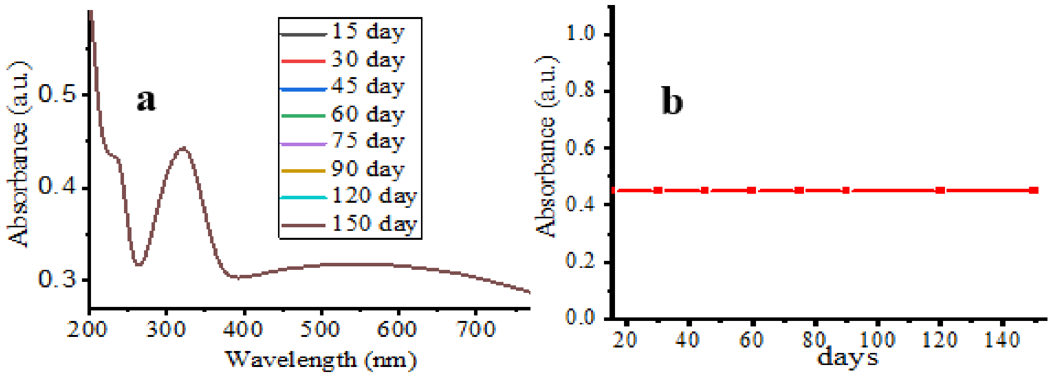

Figure S6. Stability study of saponin mediated synthesized TiO_2 NPs within five consecutive months.

Table S1. XRD of TiO_2 NPs

2 θ	Θ	FWHM	λ	K - value	d (nm)
25.5632	12.78158	0.3685	0.15406	0.9	22.10623
37.2002	18.60009	0.29492	0.15406	0.9	28.42162
38.1304	19.06521	0.29687	0.15406	0.9	28.31321
38.9147	19.45737	0.34489	0.15406	0.9	24.42944
48.3721	24.18605	0.3944	0.15406	0.9	22.08099
54.1177	27.05883	1.885	0.15406	0.9	4.732496
55.358	27.67898	1.2812	0.15406	0.9	7.001935
					**Average : 19.58 nm**

Table S2. Effects of sunlight irradiation, UV- light (254 nm), and catalyst on degradations efficiencies of both MB and MO with the absence and presence of a catalyst.

Name of module dyes	Types of irradiation light	Degradation (%)	
		Absence of TiO_2NPs	Presence of TiO_2 NPs
Methylene blue (MB)	Solar light	6.38	94
	UV (254nm)	4.56	68
	Dark Room	0	12
Methyl orange (MO)	Solar light	5.28	91
	UV (254nm)	4.7	63
	Dark Room	0	9

Table S3.a and b ANOVA analysis

a. Analysis of Variance for Quadratic model of MB.

Source	Sum of Squares	Df	Mean Square	F-value	p-value	Fit Statistics
**Model**	3305.36	14	236.10	195.28	< 0.0001	SD = 1.10
**A**	856.83	1	856.83	708.70	< 0.0001	Mean = 83.34
**B**	352.19	1	352.19	291.30	< 0.0001	Cv % = 1.32
**C**	50.18	1	50.18	41.51	< 0.0001	R^2 = 0.9949
**D**	58.39	1	58.39	48.29	< 0.0001	Adjusted R^2 = 0.9898
**AB**	1.58	1	1.58	1.30	0.2729	Predicted R^2 = 0.9726
**AC**	118.05	1	118.05	97.64	< 0.0001	Adeq precision = 42.8642
**AD**	52.56	1	52.56	43.48	< 0.0001	
**BC**	126.00	1	126.00	104.22	< 0.0001	
**BD**	125.78	1	125.78	104.03	< 0.0001	
**CD**	527.16	1	527.16	436.03	< 0.0001	
**A^2**	377.95	1	377.95	312.61	< 0.0001	
**B^2**	378.57	1	378.57	313.12	< 0.0001	
**C^2**	91.14	1	91.14	75.38	< 0.0001	
**D^2**	654.12	1	654.12	541.03	< 0.0001	
**Residual**	16.93	14	1.21	-	-	
**Lack of Fit**	15.39	10	1.54	4.00	0.0968	

b. Analysis of Variance for Quadratic model of MO.

Source	Sum of Squares	Df	Mean Square	F-value	p-value	F-statics
**Model**	752.76	14	53.77	352.68	< 0.0001	SD = 0.3905
**A**	0.7351	1	0.7351	4.82	0.0455	Mean = 78.16
**B**	35.19	1	35.19	230.83	< 0.0001	Cv % = 0.4996
**C**	82.64	1	82.64	542.01	< 0.0001	R^2 = 0.9972
**D**	311.81	1	311.81	2045.23	< 0.0001	Adjusted R^2 = 0.9943
**AB**	0.0121	1	0.0121	0.0794	0.7823	Predicted R^2 = 0.9857
**AC**	0.6400	1	0.6400	4.20	0.0597	Adeq precision = 68.9120
**AD**	18.45	1	18.45	121.00	< 0.0001	

**BC**	0.7569	1	0.7569	4.96	0.0428	
**BD**	76.48	1	76.48	501.61	< 0.0001	
**CD**	0.6320	1	0.6320	4.15	0.0611	
**A^2**	0.2471	1	0.2471	1.62	0.2237	
**B^2**	1.53	1	1.53	10.01	0.0069	
**C^2**	10.71	1	10.71	70.23	< 0.0001	
**D^2**	199.97	1	199.97	1311.62	< 0.0001	
**Residual**	2.13	14	0.1525			
**Lack of Fit**	1.78	10	0.1779	2.00	0.2634	

Table S4 a and b for both MB and MO

Model adequacies

a. Adequacies of models for MB.

Source	Sum of Squares	Df	Mean Square	F-value	p-value	Remark
		Sequential Model Sum of Squares				
Linear	1317.59	4	329.40	3.94	0.0134	
Quadratic	1036.65	4	259.16	214.36	< 0.0001	Suggested
Cubic	15.21	8	1.90	6.65	0.0165	Aliased
		Lack of Fit Tests				
Linear	2003.16	20	100.16	260.49	< 0.0001	
Quadratic	15.39	10	1.54	4.00	0.0968	Suggested
Cubic	0.1776	2	0.0888	0.2310	0.8037	Aliased
		Model Summary Statistics				
Source	Std. Dev.	R^2	Adjusted R^2	Predicted R^2	PRESS	
Linear	9.14	0.3966	0.2960	0.1623	2783.15	
Quadratic	1.10	0.9949	0.9898	0.9726	91.04	Suggested
Cubic	0.5347	0.9995	0.9976	0.9916	27.98	Aliased

b. Adequacies of models for MO.

**Source**	**Sum of Squares**	**Df**	**Mean Square**	**F-value**	**p-value**	**Remark**
		Sequential Model Sum of Squares				
Linear	430.38	4	107.59	7.96	0.0003	
Quadratic	225.42	4	56.36	369.64	< 0.0001	Suggested
Cubic	1.45	8	0.1812	1.59	0.2952	Aliased
		Lack of Fit Tests				
Linear	324.16	20	16.21	182.18	<0.0001	
Quadratic	1.78	10	0.1779	2.00	0.2634	Suggested
Cubic	0.3289	2	0.1645	1.85	0.2701	Aliased
		Model Summary Statistics				
**Source**	**Std. Dev.**	**R^2**	**Adjusted R^2**	**Predicted R^2**	**PRESS**	
Linear	3.68	0.5701	0.4985	0.3479	492.30	
Quadratic	0.3905	0.9972	0.9943	0.9857	10.80	Suggested
Cubic	0.3378	0.9991	0.9958	0.9365	47.92	Aliased

Table S5. Kinetic parameters for the degradations of MB and MO onto green synthesized TiO_2 NPs.

Kinetic model	Parameters	MB	MO
Langmuir–Hinshelwood(L-H)	K (min^{-1})	1.7 x 10^{-4}	3.2 x 10^{-4}
	R^2	0.998	0.997
Pseudo-first-order kinetics	K_1(min^{-1})	4.17x10^{-6}	3.4x10^{-5}
	q_e (mg/g)	7.85	2.225
	R^2	0.99	0.978
Pseudo-second-order kinetics	K_2 (g/mg min)	5.33x10^{-7}	-2.57x10^{-3}
	q_e (mg/g)	37	11.41
	R^2	0.98	0.95

S.Table 6. Recycling of Photocatalyst.

$$\% \text{ Dye Degradation } = \frac{A_o - A_t}{A_o} \times 100\%$$

A_o= initial absorbance of dye solutions

A_t = absorbance of dye solutions after photocatalytic degradation

	MB			MO		
**Runs Time**	A_0 initial concentration	Final concentration	Degradation %	Initial concentration	Final concentration	Degradation%
**1st**	2.57	0.2056	92	2.72	0.3264	88
**2nd**	2.57	0.257	90	2.72	0.408	85
**3rd**	2.57	0.3598	86	2.72	0.5168	81
**4th**	2.57	0.4626	82	2.72	0.625	77.9
**5th**	2.57	0.5397	79	2.72	0.68	75
